# Case report: Palliation of right pulmonary artery compression with overlapping, self-expanding vascular stents and toceranib phosphate in a dog with a large, compressive chemodectoma

**DOI:** 10.3389/fvets.2024.1398129

**Published:** 2024-09-30

**Authors:** Claudia Serrano Ferrel, Randolph L. Winter, Kara L. Maneval, Brad M. Matz, Noelle S. Bergman, Cierra Starbird, Jey Koehler, PenTing Liao

**Affiliations:** ^1^Department of Clinical Sciences, College of Veterinary Medicine, Auburn University, Auburn, AL, United States; ^2^Department of Pathobiology, College of Veterinary Medicine, Auburn University, Auburn, AL, United States

**Keywords:** angioplasty, aortic body tumor, dog, stent, interventional

## Abstract

Acquired pulmonary artery branch stenosis without main pulmonary artery involvement due to external compression by neoplasia has been described in human and veterinary medicine. Over time, this can result in right ventricular hypertension and right-sided heart failure. Endovascular stenting offers quick relief from signs, while the underlying cause is addressed. Here, we present a dog with severe right pulmonary artery compression caused by a chemodectoma, which was treated with two, overlapping, self-expanding vascular stents and chemotherapy. The patient experienced immediate symptomatic relief, progressive stent expansion over time, and has been free of clinical symptoms for 5 months post implantation.

## 1 Introduction

Chemodectomas are frequently diagnosed in the dog, as this neoplasia is the second most common cardiac neoplasm in the dog (1–6). Chemodectomas are considered slower growing tumors that have a low metastatic potential. When present, these neoplasia can cause the development of pericardial effusion, external compression to nearby vascular or cardiac structures, or right heart failure (1–6). Treatment options for large chemodectomas May include vascular stent implantation, chemotherapy with toceranib phosphate, as well as radiation therapy and surgical resection for selected cases (6, 7). When clinical signs are caused by vascular obstruction due to compression, the use of endovascular stents has been well documented as a method of relieving vascular obstruction from external compression in both veterinary and human patients (4, 8–10) Vascular stent types include those that are balloon expandable and those that are self-expanding stents (8, 9).

We report a dog with acquired right pulmonary artery stenosis and secondary syncopal episodes, both of which improved with vascular stent implantation. Self-expanding vascular stent implantation was successfully used in an overlapping stent method to immediately improve clinical signs in the dog of this report. The documented expansion of the stents over time was likely secondary to gradual stent dilation and the use of toceranib phosphate documented in the dog of this report (6). As of 5-months post-operatively, the patient has not experienced any exercise intolerance or syncope.

## 2 Clinical report

### 2.1 Case presentation and diagnostic investigations

A 9-year-old, male intact Labrador retriever dog was presented to the Emergency and Critical Service of the Auburn University Veterinary Teaching Hospital for exercise intolerance and multiple episodes of syncope of progressively increasing frequency. One day prior to presentation, the dog was evaluated by the primary care veterinarian for syncope and diagnostics revealed suspected cardiomegaly, but no additional abnormalities were observed. At presentation, cardiac auscultation revealed muffled heart sounds, mild tachypnea, and infrequent premature abnormal heart beats.

**Figure 1:**
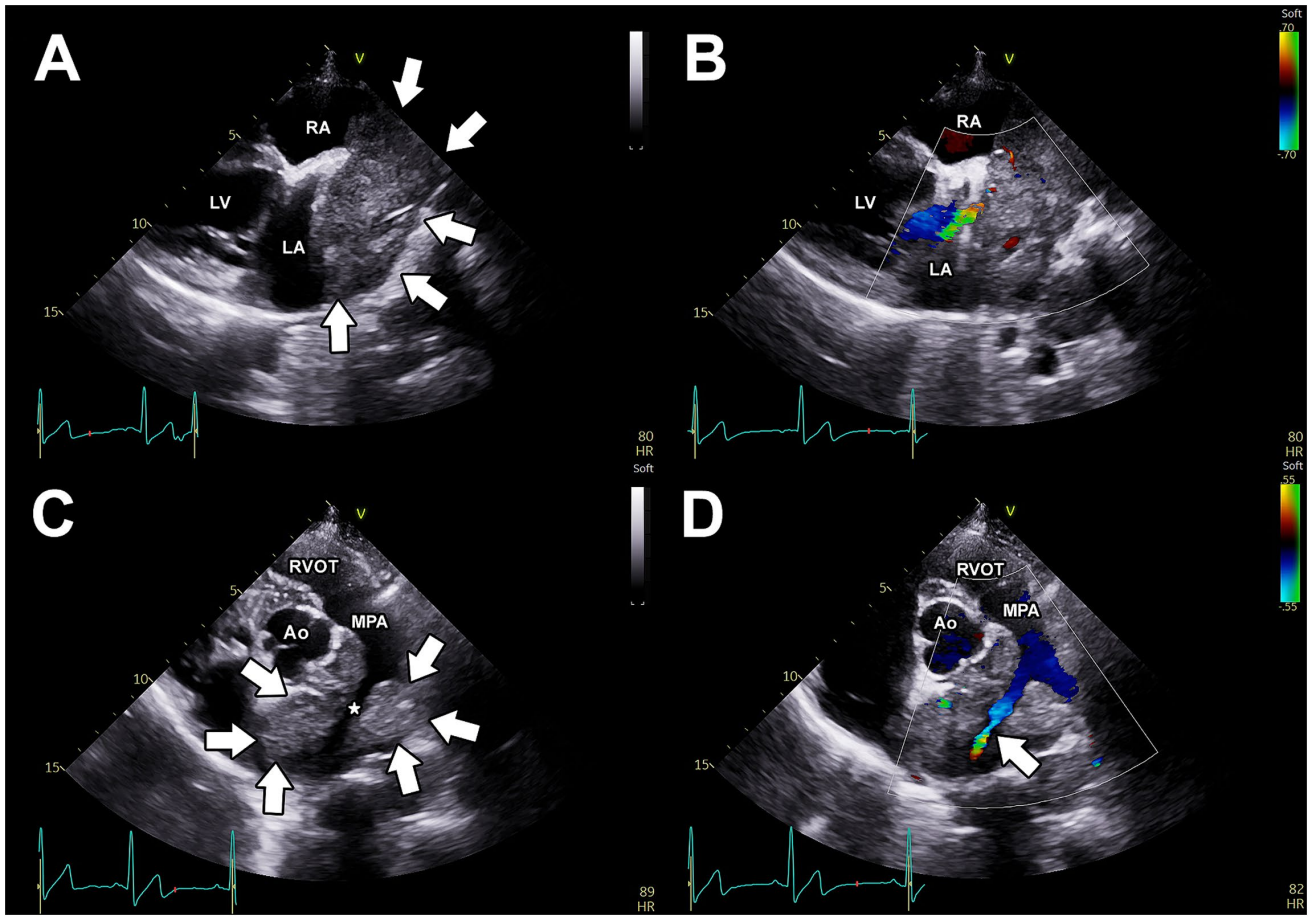
Pre-operative, transthoracic echocardiographic images. **(A)** Right parasternal long-axis view, demonstrating a large, heterogenous, irregular mass at the base of the heart. From this view, the mass is observed to be in close association with the left and right atria. Using color Doppler imaging **(B)**, partial compression of a pulmonary vein is apparent. **(C)** Two-dimensional **(C)** and color Doppler imaging **(D)** of the left parasternal short-axis view demonstrates that the mass (arrows) completely surrounds the right pulmonary artery (*) and causes obstruction to blood flow through this artery (white arrow). Ao, aorta; LA, left atrium; LV, left ventricle; MPA, main pulmonary artery; RA, right atrium; RVOT, right ventricular outflow tract.

Thoracic radiography revealed dorsal displacement of the trachea cranial to the heart and increased soft-tissue opacity in the region of the heart base, consistent with a possible heart base mass. Two-dimensional and M-mode transthoracic echocardiography revealed mild left ventricular systolic dysfunction and mild left ventricular dilation. Moderate right atrial and right ventricular dilation were observed. Color Doppler imaging revealed mild mitral and tricuspid regurgitation. A large mass of heterogeneous echogenicity, with irregular borders was observed at the base of the heart (Figure 1A). This mass was partially compressive of the left atrium, and color Doppler imaging revealed partial pulmonary venous compression (Figure 1B). This mass was also observed near the bifurcation of the main pulmonary artery (PA) and could be visualized entirely surrounding the right pulmonary artery (RPA) near its origin (Figure 1C). Color Doppler interrogation demonstrated turbulent blood flow at the origin of the RPA. Within the RPA, an extended length of compression of roughly 5 cm was observed in which delayed emptying of turbulent blood flow was observed, with the most severe focal stenosis located roughly 2.1 cm distal from the origin of the RPA (Figure 1D). For the entire length of the extended compression of the RPA, the mass was observed surrounding it. In addition to the extended length of compression, there was one focal area of worse stenosis within the RPA roughly 2–3 cm distal to the origin of the RPA. At this discrete area of stenosis, the RPA lumen measured 4 mm in diameter. For comparison, the origin of the left pulmonary artery (LPA) measured 11 mm in diameter. All of this information suggested an important and complicated stenosis of the RPA, with an extended length of compression which had a focal worsening, secondary to external compression by the heart base mass. Pericardial effusion was not observed. A simultaneous electrocardiogram during the echocardiogram revealed an underlying sinus rhythm with infrequent atrial premature complexes.

Treatment options including vascular stent implantation were discussed and based on the frequency and progressive nature of the syncopal events, a decision was made to perform RPA stent implantation immediately.

### 2.2 Treatment and clinical outcome

**Figure 2:**
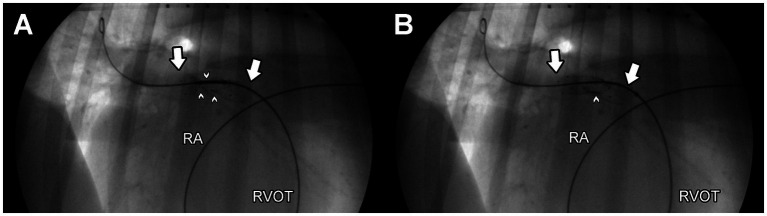
Left lateral, fluoroscopic images of the right pulmonary artery after deployment of a single endovascular stent **(A)** and after deployment of two, overlapping endovascular stents **(B)** to relieve obstruction of the right pulmonary artery. A guidewire is observed within the distal right pulmonary artery, and the point of maximal obstruction is noted by small white arrows. The length of the stent/s is notated by large white arrows. The patient’s head is to the right in these images. A marker catheter is observed in the patient’s esophagus. RA, right atrium; RVOT, right ventricular outflow tract.

The dog was given 35 mcg acepromazine IV as a pre-medication, as well as 70 mcg fentanyl IV and 35 mg lidocaine IV immediately before anesthetic induction. Anesthesia was induced with 60 mg propofol and 10 mg midazolam IV. After anesthetic induction, a continuous rate infusion of fentanyl (6 mcg/kg/h), lidocaine (50 mcg/kg/min), and dobutamine (5 mcg/kg/min) were initiated. Isoflurane in oxygen was used throughout the procedure. The patient was placed in left lateral recumbency for percutaneous access to the right external jugular vein with a 10 Fr introducer (Boston Scientific, MN, United States). A 5-Fr x 100 cm non taper angle catheter (Terumo Medical, NJ, United States) was advanced into the main pulmonary artery for selective angiography. The selective angiogram (Supplementary Figure 1A) demonstrated external compression on the RPA (~ 5–6 cm), with reducedcontrast entry into and delayed exit from the RPA compared to the LPA. Compression of the LPA was not observed. The 5-Fr catheter was advanced into the RPA across the compression, and then a 0.035″ x 260 cm super stiff guidewire (Boston Scientific, MN, United States) was placed into the distal RPA. Over this guide wire, a 9 mm x 40 mm vascular self-expanding stent (Zilver 635 Vascular self-expanding stent, Cook Medical, IN, United States) was advanced into the RPA. Successful deployment of the stent was achieved, and the fluoroscopy and subsequent selective angiography of the main pulmonary artery revealed a persistent, discrete area of residual stenosis within the deployed stent length (Figure 2A). An additional self-expanding vascular stent (10 mm x 40 mm, Zilver 635 Vascular self-expanding stents, Cook Medical, IN, United States) was then successfully deployed, centered on the point of persistent stenosis (Figure 2B).

**Figure 3:**
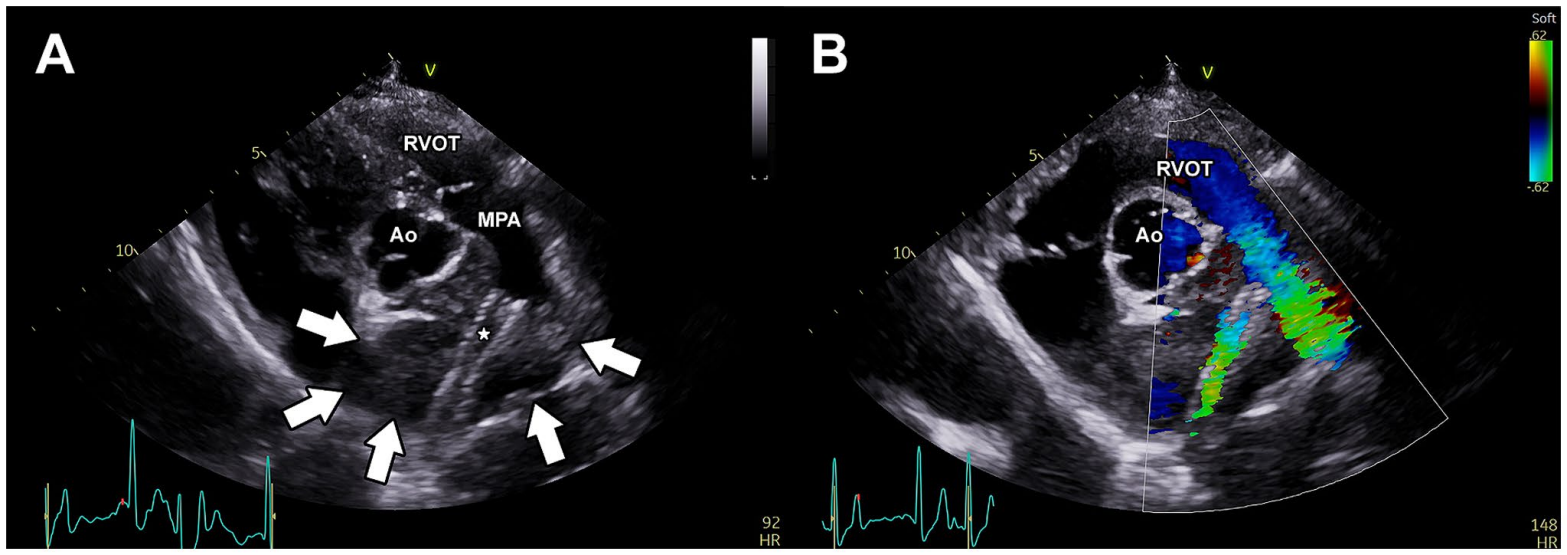
Post-operative transthoracic echocardiography of the vascular stents implanted within the right pulmonary artery branch. **(A)** Two-dimensional image of the left parasternal short-axis view demonstrating the location of the endovascular stents (*) within the right pulmonary artery. The borders of the mass are indicated by white arrows. **(B)** Color Doppler imaging of the right parasternal short-axis view demonstrating stent patency. Ao, aorta; MPA, main pulmonary artery; RVOT, right ventricular outflow tract.

**Figure 4:**
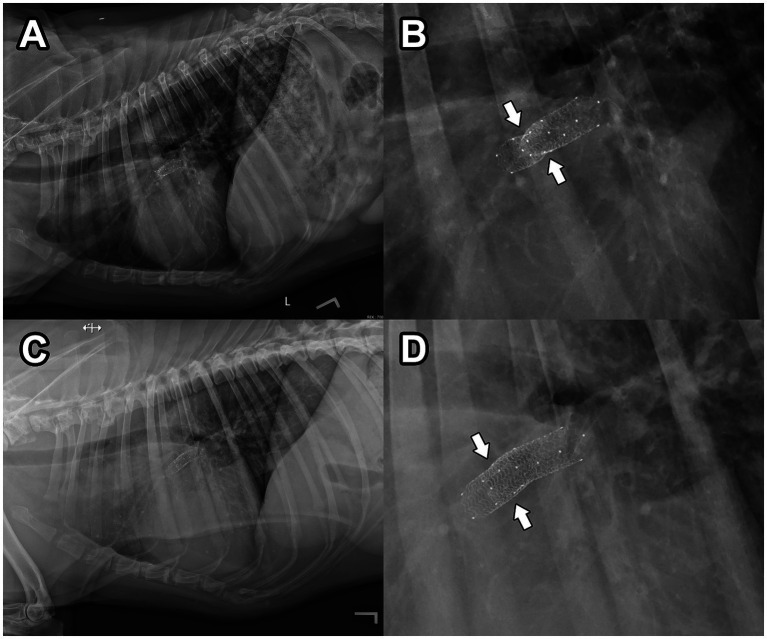
Post-operative, left lateral thoracic radiography 24 h **(A,B)** and 6 weeks **(C,D)** after endovascular stent implantation. In **(B)**, a magnified view of the radiograph presented in **(A)** reveals an area of persistent obstruction within the deployed stents (white arrows). In **(D)**, a magnified view of the radiograph presented in **(C)** reveals more complete expansion of the endovascular stents compared to **(B)**, with an area of persistent obstruction no longer observed (white arrows). The patient’s head is to the left in these images.

Repeat angiography (Supplementary Figure 1B) revealed all portions of the stent to be more fully expanded, with subjectively normal blood flow into the RPA (similar in blood flow rate of the LPA). No complications were observed and the dog recovered well from anesthesia. The anti-platelet drug clopidogrel (Bristo-Meyers Squibb, NJ, United States) was initiated at a dose of 75 mg PO BID (2.1 mg/kg) as prophylactic therapy to prevent stent thrombosis. The following day, transthoracic echocardiography revealed improved blood flow through the RPA (Figure 3) and thoracic radiography revealed stable position of the stents (Figures 4A,B). The dog was discharged with a surgical biopsy scheduled for the following week. Clinical signs including syncope were not observed during this time. An incisional biopsy was performed using a similar anesthetic protocol as during stent implantation. The mass was exposed through the mediastinum, and a 6 mm dermal punch was placed into the mass. The sample was gently grasped and the base was sharply divided with scissors. Gelatin sponge was placed in the defects. This resulted in a definitive diagnosis of chemodectoma via histopathology (Supplementary Figure 2). A percicardectomy was not performed as the mass was extrapericardial and cranial to the heart base based on the surgical perspective. Chemotherapy with toceranib phosphate (Zoetis, NJ, United States) was initiated at 100 mg (2.7 mg/kg) PO q48h after this surgery as well as pimobendan (Boehringer Ingelheim Pharmaceuticals, Inc., CT, USA) 10 mg (0.27 mg/kg) PO BID.

At an evaluation 2-months post-operatively, exercise intolerance and syncope were not reported despite vigorous exercise at home as reported by the owner. Transthoracic radiography confirmed a stable position of the stents in the RPA, and complete expansion of the stents was observed (improvement compared to the previous exam; Figures 4C,D). Transthoracic echocardiography revealed appropriate blood flow into the RPA through the vascular stents. The heart base mass was subjectively smaller in size compared to the previous exam. Toceranib phosphate and pimobendan were continued at the previously described dosages, and as of 5-months post-operatively, the patient has not experienced any exercise intolerance or syncope.

## 3 Discussion

Chemodectomas are the second most common cardiac neoplasm in dogs (6). Chemodectomas are generally considered slow-growing tumors with low metastatic potential and May be diagnosed as an incidental finding. Development of pericardial effusion or external compression to nearby vascular or cardiac structures May cause clinical signs in dogs (1–6). The dog of this report did not have pericardial effusion but did have acquired RPA stenosis secondary to compression by the large chemodectoma. These clinical symptoms fully resolved after placing two overlapping vascular stents across the stenotic lesion. In addition to vascular stent implantation, treatment options for chemodectoma in the dog include chemotherapy with toceranib phosphate, as well as radiation therapy and surgical resection for selected cases (6, 7). Clinical signs were immediately improved after stent implantation, and gradual stent dilation at the point of stenosis was documented over a period of 2 months. The combination of overlapping self-expanding stent implantation and the administration of an anti-proliferative and anti-angiogenic chemotherapeutic agent are likely responsible for the progressive stent dilation.

Acquired pulmonary artery branch stenosis secondary to heart base masses like chemodectomas can lead to right ventricular hypertension and subsequent clinical signs such as collapse or right sided congestive heart failure (CHF) (4). Although the dog of this report never developed right sided CHF, there were echocardiographic findings suggestive of elevated right heart pressure including moderate right atrial and right ventricular dilation. In this case, there was a nonuniform and diffuse narrowing at the origin of the RPA and throughout its course (~ 55 mm), with a maximal lumen reduction of 64% when compared to the normal LPA branch. This stenotic lesion was classified as Type 1 B (a single elongated stenosis confined to the RPA) according to the classification of congenital stenosis of the PA branches (11). Advanced imaging modalities such as computed tomography with angiography or magnetic resonance imaging could be utilized for similar cases, as these modalities May provide more information regarding the anatomy and blood supply of a compressive neoplastic lesion. Advanced imaging was refused by the owner in this case due to financial constraints. The complexity of the stenosis in combination with the progressive clinical symptoms prompted the immediate transcatheter intervention of endovascular stent implantation.

Implantation of endovascular stents has been documented as a method of managing acquired PA stenosis secondary to external compression in both veterinary and human patients (2–4, 8–10) Vascular stent types include balloon expandable and self-expanding stents (8, 9). Balloon expandable stents are made of stainless steel, are rigid, and May display chronic recoil over time. Alternatively, self-expanding stents are made of nitinol which provides enhanced flexibility and chronic expansion without recoil over time. These characteristics make them ideal for stenotic lesions in superficial vessels or in anatomic regions where external compression forces by adjacent structures are persistent (1, 12). In the dog of this report, self-expanding vascular stent implantation was chosen in order to ease access across the stenosis and to minimize stent recoil over time given the persistent external compression of the RPA. In fact, expansion of the stents diameter over time was documented in the dog of this report based on radiographic imaging. The diameter of the stents were chosen because ~9–10 mm approximated the diameter of the LPA at its origin as observed on selective angiography. Once the 9 mm stent was implanted, the larger size 10 mm diameter stent was chosen so as to avoid possible stent migration over time.

The dog of this report had acquired RPA stenosis and secondary syncopal episodes, both of which improved with vascular stent implantation. Clinical improvement after vascular stent implantation for acquired RPA stenosis has been documented in dogs and in humans (1–4, 8–10). In one case, an adult dog with chylothorax secondary to external compression by a mass, and self-expanding vascular stents were implanted in the RPA and cranial vena cava to relieve this obstruction (2). Balloon-dilatable vascular stents have also been utilized to relieve pulmonary artery branch stenosis in dogs and in human patients (1, 3, 8, 9). In the dog of this report, a self-expanding stent was used due to the length and tortuous course of the stenosis. However, after a single stent implantation, a selective angiography of the main pulmonary artery revealed persistent external compression. The addition of the second stent acted to increase the combined radial force of the overlapping stents and therefore more completely dilate the RPA to restore normal blood flow. An immediate increase in the stent diameter was observed in the dog of this report with implantation of the second stent, and this effect was increased even further over the course of 2 months.

Overlapping self-expandable stents is a technique used for multiple or elongated stenoses, persistent stenosis after single stent implantation, or in response to stent migration away from the target lesion (10). In human patients with overlapping stent implantation, short and intermediate-term complications are not observed (10). Placement of overlapping self-expanding stents May be more complicated than a single stent placement due to stent-to-stent interaction and overall less predictable deployment (10, 13). Compared to single stent implantation, overlapping stent implantation May also have an increased risk of chronic inflammation and subsequent thrombosis locally (13, 14). We did not observe thrombosis post-operatively in the dog of this report, which May in part be due to the prophylactic anti-thrombotic medication that was initiated. When persistent stenosis remains despite single stent implantation remains, another option to consider is dilation of the implanted stent with a balloon catheter. Dilation of a self-expanding stent has been documented with balloon catheter made of thin, compliant material, and over-dilation of a self-expanding stent with a balloon catheter has also been documented (12, 15). However, dilation of a self-expanding stent after implantation risks stent embolization or fracture (12).

In conclusion, endovascular stent implantation is an effective aspect of acute treatment for acquired PA branch stenosis secondary to external compression by heart base masses. Self-expanding vascular stents May be preferable over balloon expandable vascular stents for elongated and tortuous peripheral stenoses due to their intrinsic flexibility properties. Successful transcatheter stent implantation May acutely relieve the symptoms of stenosis while further treatment like chronic chemotherapy and radiation therapy May help address persistent problems over a longer time period.

## Data Availability

Requests to access the datasets should be directed to rlw0041@auburn.edu.

## Supplementary material

SUPPLEMENTARY FIGURE 1 Left lateral, selective angiography of the main pulmonary artery with a 5 Fr angiographic catheter prior to **(A)** and following **(B)** deployment of overlapping endovascular stents. In **(A)**, the impingement by the mass (arrows) and obstruction to blood flow is apparent in the right pulmonary artery as contrast slowly moves past the obstruction and is relatively concentrated at the point of obstruction. In **(B)**, contrast readily moves through the right pulmonary artery at a similar rate compared to the unobstructed left pulmonary artery. The tip of the catheter is marked with a (*), and the entire length of the overlapping stents is marked by large white arrows. The patient’s head is to the right in these images. A marker catheter is observed in the patient’s esophagus. RA, right atrium; RV, right ventricle.

SUPPLEMENTARY FIGURE 2 Aortic body tumor (chemodectoma), dog. Polygonal to round cells are arranged in packets in a delicate well-vascularized stroma. Cells have abundant granular cytoplasm, round nuclei with finely stippled chromatin and one to multiple nucleoli, and a variable rim of smaller basophilic cells with scant cytoplasm (sustentacular cells). Anisocytosis and anisokaryosis are moderate. Hematoxylin and eosin stain.

## References

[1] Zablah JE Morgan GJ. Pulmonary artery stenting. Interv Cardiol Clin. (2019) 8:33–46. doi: 10.1016/j.iccl.2018.08.005 30449420

[2] Taylor S Rozanski E Sato AF Rush JE. Vascular stent placement for palliation of mass-associated chylothorax in two dogs. J Am Vet Med Assoc. (2017) 251:696–701. doi: 10.2460/javma.251.6.696, PMID: 28857703

[3] Bussadori CM Claretti M Borgonovo S Boz E Papa M Rossi C. Branch pulmonary artery stent placement in a dog with heart base neoplasia. J Vet Cardiol. (2020) 30:17–22. doi: 10.1016/j.jvc.2020.05.002 32619933

[4] Batra K Saboo SS Kandathil A Canan A Hedgire SS Chamarthy MR. Extrinsic compression of coronary and pulmonary vasculature. Cardiovasc Diagn Ther. (2021) 11:1125–39. doi: 10.21037/cdt-20-155, PMID: 34815964 PMC8569266

[5] Coto GM Musser ML Tropf MA Ward JL Seo YJ Mochel JP. A multi-institutional retrospective analysis of Toceranib phosphate for presumed or confirmed canine aortic body Chemodectomas. Front Vet Sci. (2021) 8:e635057. doi: 10.3389/fvets.2021.635057, PMID: 33614771 PMC7892462

[6] Aupperle H Marz I Ellenberger C Buschatz S Reischauer A Schoon HA. Primary and secondary heart tumors in dogs and cats. J Comp Pathol. (2007) 136:18–26. doi: 10.1016/j.jcpa.2006.10.002, PMID: 17270204

[7] Lew FH McQuown B Borrego J Cunningham S Burgess KE. Retrospective evaluation of canine heart base tumours treated with toceranib phosphate (Palladia): 2011-2018. Vet Comp Oncol. (2019) 17:465–71. doi: 10.1111/vco.12491, PMID: 31069932

[8] Trivedi KR Benson LN. Interventional strategies in the management of peripheral pulmonary artery stenosis. J Interv Cardiol. (2003) 16:171–88. doi: 10.1046/j.1540-8183.2003.08031.x, PMID: 12768922

[9] Krisnanda C Menahem S Lane GK. Intravascular stent implantation for the management of pulmonary artery stenosis. Heart Lung Circ. (2013) 22:56–70. doi: 10.1016/j.hlc.2012.08.008, PMID: 23017591

[10] Jang GY Ha KS. Self-expandable stents in vascular stenosis of moderate to large-sized vessels in congenital heart disease: early and intermediate-term results. Korean Circ J. (2019) 49:932–42. doi: 10.4070/kcj.2019.0067, PMID: 31190478 PMC6753030

[11] Franch RH Gay BB. Congenital stenosis of the pulmonary artery branches. A classification, with postmortem findings in two cases. Am J Med. (1963) 35:512–29. doi: 10.1016/0002-9343(63)90149-9 14072375

[12] Duerig TW Wholey M. A comparison of balloon- and self-expanding stents. Minim Invasive Ther Allied Technol. (2002) 11:173–8. doi: 10.1080/136457002760273386 16754067

[13] Rikhtegar F Wyss C Stok KS Poulikakos D Müller R Kurtcuoglu V. Hemodynamics in coronary arteries with overlapping stents. J Biomech. (2014) 47:505–11. doi: 10.1016/j.jbiomech.2013.10.048, PMID: 24275438

[14] Grenadier E Shofti R Beyar M Lichtig H Mordechowitz D Globerman O. Self-expandable and highly flexible nitinol stent: immediate and long-term results in dogs. Am Heart J. (1994) 128:870–8. doi: 10.1016/0002-8703(94)90582-7, PMID: 7942477

[15] Koethe Y Lokken RP Lehrman ED Kerlan RK Roberts JP Rhee SJ. Overdilation of a 6-mm self-expanding stent with a 10-mm balloon-expandable stent graft preserves failing Meso-rex bypass. J Vasc Interv Radiol. (2020) 31:521–3. doi: 10.1016/j.jvir.2019.09.024, PMID: 32007411

