# Supplementary figures and images for: Case report: Palliation of right pulmonary artery compression with overlapping, self-expanding vascular stents and toceranib phosphate in a dog with a large, compressive chemodectoma
Source: Front Vet Sci. 2024 Sep 30;11:1398129. doi: 10.3389/fvets.2024.1398129 (PMC11471735; doi:10.3389/fvets.2024.1398129)

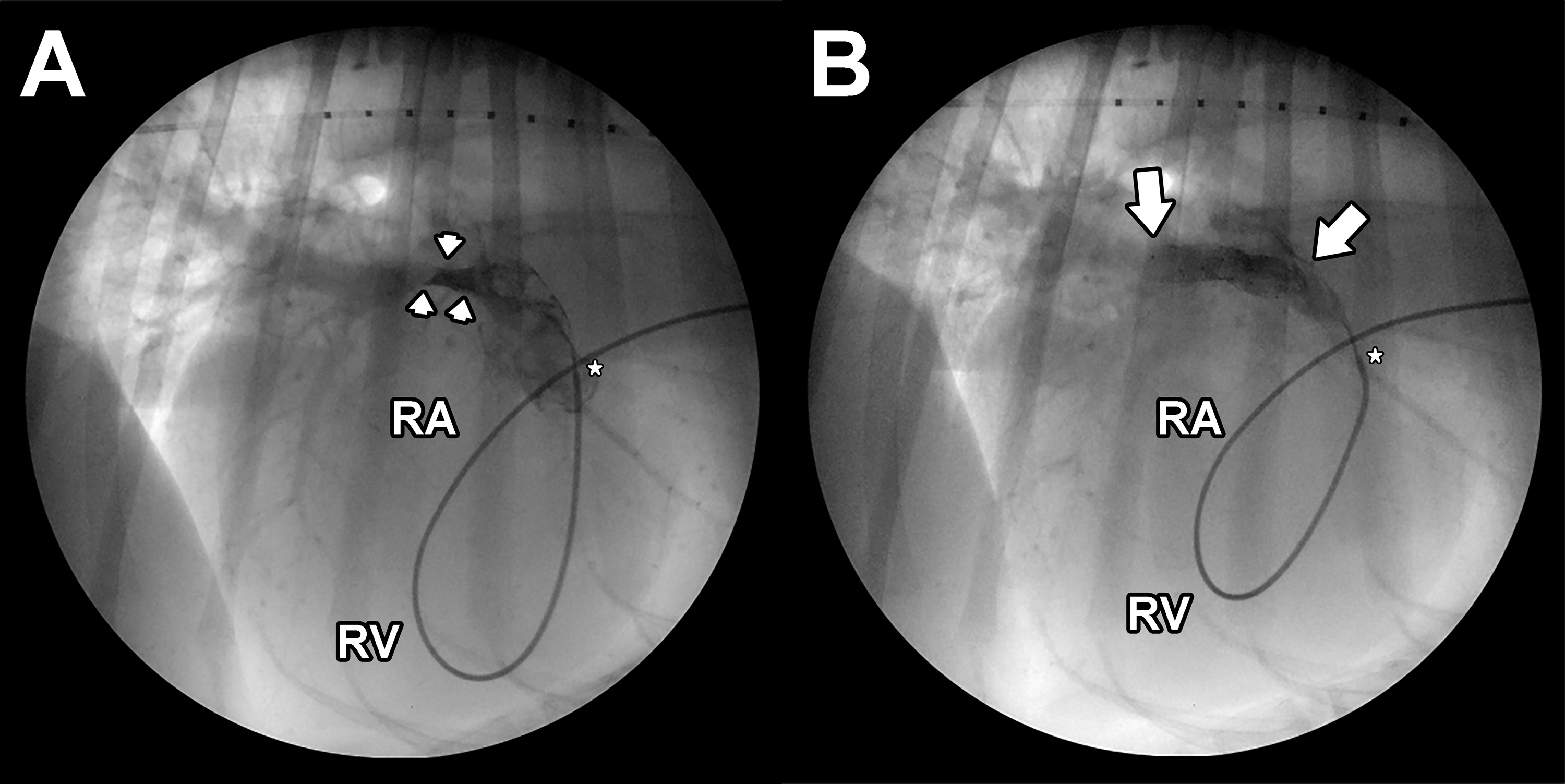

Supplement: SUPPLEMENTARY FIGURE 1 — Left lateral, selective angiography of the main pulmonary artery with a 5 Fr angiographic catheter prior to (A) and following (B) deployment of overlapping endovascular stents. In (A), the impingement by the mass (arrows) and obstruction to blood flow is apparent in the right pulmonary artery as contrast slowly moves past the obstruction and is relatively concentrated at the point of obstruction. In (B), contrast readily moves through the right pulmonary artery at a similar rate compared to the unobstructed left pulmonary artery. The tip of the catheter is marked with a (*), and the entire length of the overlapping stents is marked by large white arrows. The patient’s head is to the right in these images. A marker catheter is observed in the patient’s esophagus. RA, right atrium; RV, right ventricle. [file Image_1.TIF]

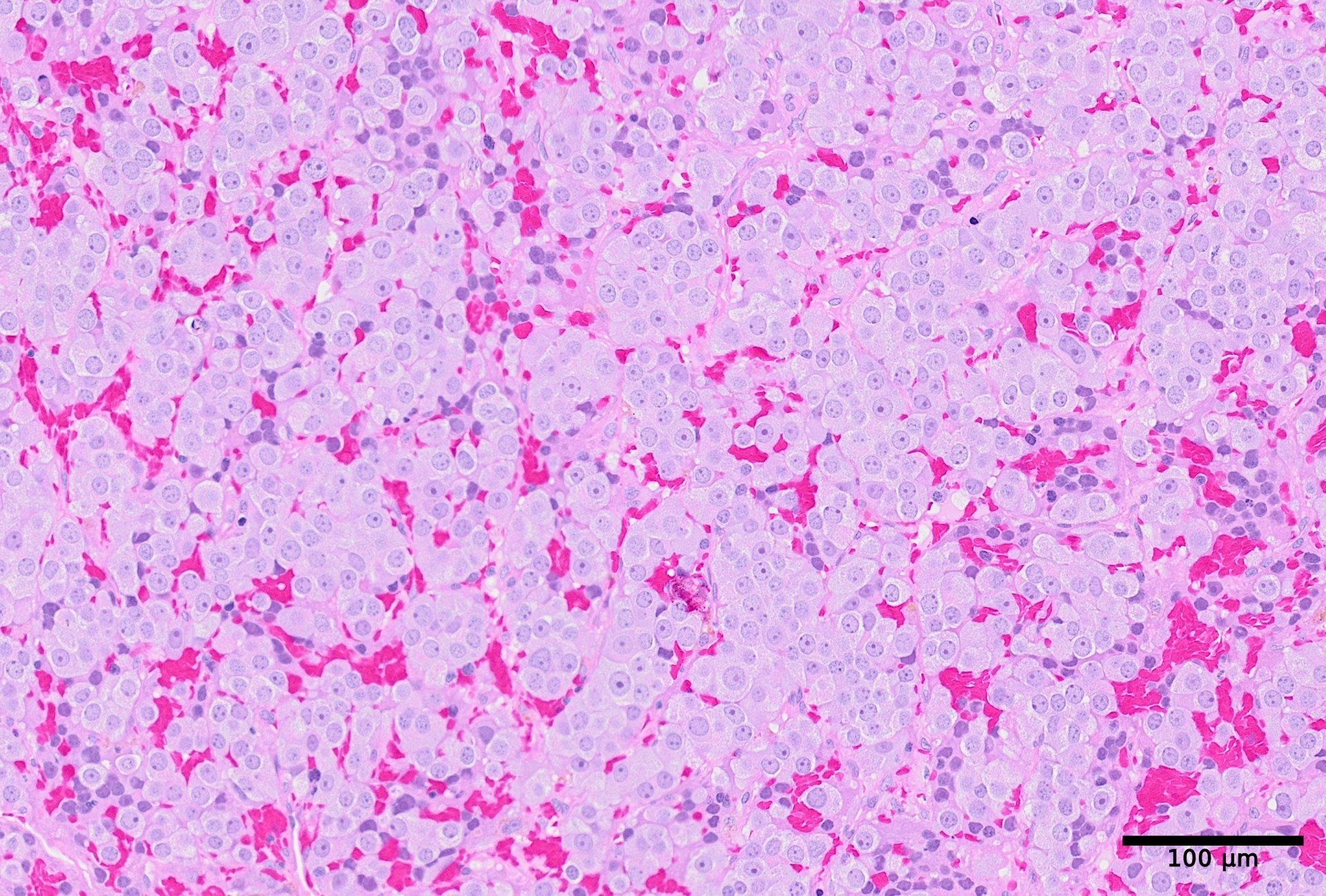

Supplement: SUPPLEMENTARY FIGURE 2 — Aortic body tumor (chemodectoma), dog. Polygonal to round cells are arranged in packets in a delicate well-vascularized stroma. Cells have abundant granular cytoplasm, round nuclei with finely stippled chromatin and one to multiple nucleoli, and a variable rim of smaller basophilic cells with scant cytoplasm (sustentacular cells). Anisocytosis and anisokaryosis are moderate. Hematoxylin and eosin stain. [file Image_2.JPEG]
